# Association of the polymorphisms *P53* gene with juvenile idiopathic arthritis in children Russian Fedration

**DOI:** 10.1186/1546-0096-9-S1-P282

**Published:** 2011-09-14

**Authors:** Alexey N Kozhevnikov, Michael V Moskalenko, Nina A Pozdeeva, Margarita F Dubko, Valentina I Larionova, Gennagy A Novik

**Affiliations:** 1 Saint-Petersburg Pediatric Medical Academy, Saint-Petersburg, Russian Federation; 2 Saint-Petersburg Research Pediatric Orthopedic Institute n. a. G.I. Turner, Saint-Petersburg, Russian Federation; 3 Saint-Petersburg State University, Department Genetics & Breeding. Saint-Petersburg, Russian Federation

## Background

Juvenile idiopathic arthritis – is a chronic systemic autoimmune disease that is a characterized by articular lesion with synovial hyperplasia and cellular infiltration. *Arg72Pro* (4ex) and *ins/del*16bp (3in) polymorphisms are associated with affects the functional activity of the p53 protein. (P.Dumont et al, 2003)

## Aim

The purpose of our study is estimate course and outcomes of juvenile idiopathic arthritis of the children with various genotypes of *p53*.

## Methods

We examined 58 children with juvenile idiopathic arthritis. Clinical, serological and x-ray manifestations were analyzed in children and correlated with the genotypes. For detection erosion bone process we used ultrasound, x-ray, MRI and diagnostic arthroscopy with synovial biopsy. We investigated (PCR-RFLP) the status of *p53 gene* this children with juvenile idiopathic arthritis and 100 healthy children living in Russian Federation.

## Results

Genotypes distributions of *Arg72Pro* and *ins/del*16bp polymorphisms did not differ significantly (p>0,05) between JIA patients and controls. Children with mild form oligo-, polyarthritis JIA achieved remission had significantly higher percentage genotype *Arg/Arg*+*del/del* compared children with severe oligo, polyarthritis duration more 5 years (89,7 vs 23,8%, p<0,01). Young girls with severe oligoarthirits, ANA-positive and erosion joint process had a significantly higher percentage of genotype *Arg/Pro*+*ins/del* compared children with mild form oligoarthritis, ANA-negative (87,5 vs 9%, p<0,01). Girls with severe polyarthritis DAS44 4.0±1.1 had significant high percentage genotype *Arg/Pro*+*ins/del* compared children with mild form polyarthritis DAS44 2.4±0.9 (67 vs 9%, p<0,01).

## Conclusion

Girls with genotype *Arg/Pro*+*ins/delp53* had more severe and aggressive form oligo-polyarthritis manifested by erosion process.

